# “You know how men are”: the gendered nature of support during pregnancy in South Africa – an exploratory convergent mixed-method study

**DOI:** 10.1080/26410397.2026.2689806

**Published:** 2026-06-17

**Authors:** Catriona Ida Macleod, Ulandi du Plessis, Elron Fouten

**Affiliations:** a Distinguished Professor and SARChI Chair, Critical Studies in Sexualities and Reproduction, Rhodes University, Makhanda, South Africa.; b Senior Researcher, Critical Studies in Sexualities and Reproduction, Rhodes University, Makhanda, South Africa; c Senior Lecturer and Head of Department, Department of Psychology, Rhodes University, Makhanda, South Africa

**Keywords:** pregnancy, support, men, fatherhood, gender, reproduction

## Abstract

Male partner support during pregnancy has been linked to positive outcomes and can enhance women’s rights to reproductive health and well-being. Such involvement can, however, have gendering effects. In addition, the role of other men in pregnancy support is under-researched. In an exploratory convergent mixed methods study, we investigated the gender dynamics evident in pregnant women's reporting of, and stories concerning, support from male partners and other significant men. We administered the Pregnancy Supportability Research Kit in two health districts of the Eastern Cape, South Africa. The closed-ended information questionnaire component was completed by 558 women, and open-ended information narrative interviews were conducted with 40 women. In the quantitative data, 12.1% of women indicated that they received no support from their partners, which, in the qualitative data, was reportedly underpinned by abandonment, paternity denial and abuse. Just under one in five (18.7%) reported that their partners were somewhat supportive. Qualitative data revealed men reluctantly taking on feminised tasks such as cooking, judgementally sharing pregnancy information, and providing support inconsistently. Of the 69.2% reporting supportive partners, emotional support was mentioned most frequently. In relation to support provided by significant others – people living in the same household, family members living outside the household, and friends – women were reported as significantly more likely to provide support than men. These findings point to the importance of nuanced understandings of the gendered dynamics in men’s support during pregnancy, particularly in programmes that engage men in confronting and undermining negative gendered norms. *DOI: 10.1080/26410397.2026.2689806*

## Plain language summary

We investigated pregnant women’s perceptions of the support they received from men close to them, including partners. Findings show that just over one out of ten women feel their partners provide no support. This could be because of men denying paternity, abandoning or abusing their partners. One in five women felt their partners provided some support. Support might be irregular or judgemental. Sometimes it was temporary, like cooking only during the pregnancy. Seven out of ten women said their partners were very supportive, especially emotionally. Study participants said that, in general, women were more likely than men to provide support. This was true whether they lived in the same household or not, and whether they were friends. As good support can positively influence pregnancy health outcomes, programmes should engage men in confronting and undermining negative gendered norms.

## Résumé

Le soutien du partenaire masculin pendant la grossesse est associé à des effets positifs et peut renforcer les droits des femmes à la santé reproductive et au bien-être. Cette participation peut néanmoins avoir des répercussions liées au genre. De plus, le rôle d’autres hommes dans le soutien à la grossesse ne fait pas l’objet de suffisamment de recherches. Dans une étude exploratoire convergente à méthodes mixtes, nous avons examiné les dynamiques de genre qui apparaissent dans les témoignages et les récits de femmes enceintes concernant le soutien de leur partenaire masculin et d’autres hommes importants pour elles. Nous avons administré le kit de recherche sur le soutien à la grossesse dans deux districts sanitaires de la province du Cap oriental, en Afrique du Sud. Le questionnaire à questions fermées a été rempli par 558 femmes et des entretiens narratifs à questions ouvertes ont été menés auprès de 40 femmes. Les données quantitatives révèlent que 12.1 % des femmes n’ont reçu aucun soutien de leur partenaire, ce qui, selon les données qualitatives, est souvent lié à l’abandon, au déni de paternité et aux mauvais traitements. Un peu moins d’une femme sur cinq (18.7 %) a déclaré que son partenaire lui prodiguait un certain soutien. Les données qualitatives ont montré que les hommes avaient du mal à assumer des tâches traditionnellement féminines comme la cuisine, partageaient les informations sur la grossesse de manière critique et offraient un soutien irrégulier. Parmi les 69,2% de femmes ayant déclaré que leur partenaire les soutenait, le soutien psychologique était le plus fréquemment mentionné. S’agissant du soutien apporté par des proches – personnes vivant sous le même toit, membres de la famille vivant hors du foyer et amis – les femmes étaient sensiblement plus susceptibles de prodiguer un soutien que les hommes. Ces résultats soulignent l’importance d’une compréhension nuancée des dynamiques de genre dans le soutien masculin pendant la grossesse, en particulier dans les programmes qui incitent les hommes à confronter et remettre en question les normes de genre négatives.

## Resumen

El apoyo de una pareja de sexo masculino durante el embarazo se ha relacionado con resultados positivos y puede mejorar los derechos de las mujeres a la salud y al bienestar reproductivos. Sin embargo, tal participación puede tener efectos de género. Además, no se ha investigado lo suficiente el papel que desempeñan otros hombres en apoyar el embarazo. En un estudio exploratorio de métodos mixtos convergente, investigamos las dinámicas de género evidentes en los relatos e historias de mujeres embarazadas sobre el apoyo que recibieron de sus parejas de sexo masculino y de otras personas importantes en su vida. Administramos el Kit de Investigación sobre el Apoyo Durante el Embarazo en dos distritos de salud de la provincia del Cabo Oriental, en Sudáfrica. Se realizaron entrevistas narrativas abiertas con 40 mujeres y 558 mujeres llenaron un cuestionario de preguntas cerradas. En los datos cuantitativos, el 12.1% de las mujeres indicaron que no recibieron ningún apoyo de su pareja, sino, según los datos cualitativos, abandono, negación de paternidad y abuso. Un poco menos de una de cada cinco mujeres (18.7%) informaron que su pareja fue algo solidario. Los datos cualitativos revelaron que los hombres asumieron de mala gana tareas feminizadas como cocinar, divulgaron información sobre el embarazo de manera crítica y brindaron apoyo irregularmente. Entre el 69.2% de mujeres que informaron tener parejas solidarias, el apoyo emocional fue mencionado con mayor frecuencia. Con relación al apoyo brindado por personas importantes –personas que viven en el mismo hogar, miembros de la familia que viven fuera del hogar y amistades– las mujeres fueron reportadas como considerablemente más propensas a brindar apoyo que los hombres. Estos hallazgos señalan la importancia de comprensiones matizadas de las dinámicas de género en el apoyo que brindan los hombres durante el embarazo, en particular en programas dirigidos a los hombres para confrontar y socavar las normas de género negativas.

## Introduction

Since the 1970s, scholars have noted (cisgender, heterosexual) men’s* increasing involvement in childbirth, childrearing and, more recently, pregnancy.^1,2^ The field of fatherhood studies is, by now, well established,^3–5^ and there is an emerging field of research on men's involvement in maternal healthcare.^6–9^ This research reflects a growing number of global health initiatives that encourage men's involvement in pregnancy, childbirth and childrearing practices (see, for example, MenEngage Africa and UNICEF's MenCare).^10^ In South Africa, where we conducted this research, Sonke Gender Justice facilitates the MenCare initiative aimed at promoting men’s involvement as fathers and caregivers in equitable, responsive and non-violent ways. These programmes have demonstrated effectiveness in facilitating positive changes in gender-equitable attitudes amongst young men.^11^

In its recommendations on health promotion interventions for maternal and newborn health, the World Health Organisation (WHO)^12^ advises that interventions should promote the involvement of men during pregnancy, childbirth and after birth. Such involvement is viewed by the WHO as potentially bolstering pregnant women’s right to reproductive health and well-being through facilitation of improved self-care, use of skilled care during pregnancy, childbirth and the postnatal period, and increased timely use of obstetric and newborn facility care. A caveat is added that such interventions should be “implemented in a way that respects, promotes and facilitates women’s choices and their autonomy in decision-making” (p. 3). In other words, the rights of women to bodily autonomy, as well as reproductive health and well-being, are emphasised.

This focus has generally been welcomed, as patriarchal gender roles that delineate women solely as carers and homemakers and men as breadwinners are oppressive and untenable from both an egalitarian and a human rights point of view.^13^ There are, nevertheless, some critiques. Hunter et al.^14^ argue that the “caring masculinity” represented in increased father involvement is not necessarily distinct from hegemonic forms of masculinity. They indicate, on the one hand, that father love and involvement are, in fact, not new. While some behaviours and expressions of care may change, others “remain the same, albeit in different guises” (p. 8). On the other hand, they argue that the uncritical uptake of notions of the “new father” may place unrealistic expectations on men within the structural and economic factors in which they are located. Along this latter line of thinking, scholars have indicated that dominant theories of caregiving and fatherhood are based in middle-class, Anglo-centred settings, whereas non-hegemonic, marginalised father groups have remained under-theorised.^15^

Much of the research on men’s involvement in maternal health (in particular) has focussed on childbirth. For example, the review commissioned by the World Health Organisation on male involvement in Maternal, Newborn and Child Health^16^ concentrated on childbirth and newborn health, not on pregnancy. In this paper, we foreground pregnancy specifically.

### Men’s involvement or support during pregnancy

Evidence suggests that a male partner's involvement during pregnancy can positively influence health outcomes for the woman and their children, thereby enhancing women’s right to reproductive health and well-being.^17–19^ The pathways to this influence include improved antenatal care attendance, skilled birth attendance, facility birth, postpartum care, birth and complications preparedness, and maternal nutrition.^20^ Perceived challenges to greater partner involvement in antenatal care include sociocultural norms, the physical layout of clinics, and health worker workloads and attitudes.^6,21^

Some researchers have, however, cautioned against an uncritical encouragement of partner involvement in pregnancies. Increased involvement can have gendered effects: for instance, men dominating decision-making about pregnancy nutrition and infant care.^20^ In their research conducted in Ghana,^22^, they found that women were negatively disposed towards the involvement of men, partially because of “fears that men's involvement may turn hitherto secure social spaces for women into insecure ones” (p. 195).

The conceptualisations of male partner involvement during pregnancy have also been critiqued. McLean,^23^ for example, argues that the “narrow” and “largely Western” definition of male involvement as an engagement with maternity services casts African men in a bad light. It restricts involvement to instrumental, clinic-based behaviours, “biomedicalizing acceptable male behaviour, and as a result, discount[ing] other types of involvement that may be equally, if not more, socially meaningful” (p. 2). In contrast, our research surfaces women’s perceptions of men’s support in the totality of their lives.

Additionally, little research has concentrated on fathers’ or men's (in general) *support* for pregnant women. Research has tended to focus on fathers’ *experiences* of pregnancy^24^ and fathers’ *involvement* in pregnancy.^25,26^ While understanding fathers’ experiences or involvement in pregnancy could be important in understanding the support they provide, experience or involvement are not the same thing as support.

When partner pregnancy *support* is foregrounded, activities outside antenatal care tend to be highlighted, as illustrated in two studies conducted in Nigeria. In John-Akinola et al.'s study,^27^ women saw social support from their male partners mainly as assisting with household chores and caring for other children. In Babatunde et al.'s study,^28^ women highlighted financial support throughout pregnancy and instrumental support (household chores and childcare) in the later stages of pregnancy. In both studies, the partner was not expected to accompany the woman to the clinic for antenatal care visits, with long waiting times being cited as a reason.

In this paper, we outline pregnant women's responses regarding the support they received during their pregnancy, focusing specifically on the gendered dimension thereof. We include support provided by other significant men, specifically men living in the same household (probably, but not necessarily, family), male family members living outside the household, and male friends. We compare this to the support provided by significant women in the same categories. This is a correction to the fact that pregnancy support provided by men other than the biological father or current partner has received little attention. Social fathers are men who provide care and support to children who are not their biological offspring nor officially adopted. In other words, these are men who act as “father figures”. Additionally, given the role that social fathers play in various contexts,^29–31^ including South Africa, the inclusion of these men in a study on pregnancy support is important.

We foreground the pregnant woman’s experience of support, as fathers’ and other men's *involvement* in pregnancy may or may not be experienced as *supportive* by the woman. As noted earlier, men’s involvement can result in the undermining of women’s rights to bodily autonomy, reproductive health and well-being.

## Methods

### Context

Our study took place within the Eastern Cape, South Africa, where unemployment levels are high, estimated at 40.1% in 2024. Men have a marginally higher rate of unemployment at 42.2% than women at 39.6%.^32^ Women tend, however, to be employed in lower-paid professions and earn, on average, 80% of what men earn.^33^ Poverty headcounts are, on average, about four percentage points lower for men than for women living below the lower-bound poverty line. In 2023, women constituted 53.6% of the poor population, and men 46.4% (Statistics South Africa).^34^ These stark statistics provide insight into the context for support during pregnancy, with women often having to rely on men – their partner or another male family member – for financial support.

### Research design and question

Using an exploratory convergent mixed method design,^35^ we posed the following research questions: What gender dynamics appear in women's reporting of and stories concerning support received during their pregnancy? As such, our study concentrates on perceived support. We understand gender dynamics as the behavioural and verbal interactions between and among men, women, and different gender groups that are underpinned by gendered power relations.

Table 1 outlines our implementation matrix for our research design – exploratory convergent mixed methods. The data supporting this study's findings are available from the corresponding author, CIM, upon reasonable request.

**Table 1.** Implementation matrix

	Quantitative component	Qualitative component
Research question	What gender dynamics appear in women's *reporting of male suppor*t (emotional, physical, material and informational) received during their pregnancy?	What gender dynamics appear in women's *stories concerning male support* received during their pregnancy?
Type of data	Questionnaire based on supportability framework	Narrative interviews
Participants	558 pregnant women; age range 14 to 46, mean age of 27.8; 11.8% in the first, 35.3% in the second and 52.8% in the third trimester of pregnancy	40 pregnant women; age range 18 to 38, mean age of 26.5 years; Five in the first, 18 in the second, and 17 in the third trimester of pregnancy
Location	Twelve antenatal clinics in two health districts	Twelve antenatal clinics in two health districts

### Research instruments

The study ran from 1 August 2021 to 31 July 2023. The quantitative and qualitative data were collected at the same time (i.e. convergently), using the Pregnancy Supportability Research Kit (PSRK). A team of researchers at the CSSR (Critical Studies in Sexualities and Reproduction research unit) developed the PSRK from the supportability framework outlined by Macleod.^36^ It is a mixed method, multi-faceted tool aimed at surfacing (1) pregnancy supportability (defined as the capacity of a woman to carry a pregnancy resulting in positive health and welfare outcomes for herself and the infant), (2) micro-level support from partner, family, friends, colleagues, healthcare providers, teachers, and religious peers, and (3) macro-level structural and norm support. The Kit was developed and refined through thorough literature searches on conceptualisations of pregnancy support, cognitive interviewing,† ^37^ and repeated workshops in which the researchers interrogated the items using the literature searchers, and results from the cognitive interviewing.

The data we report on in this paper were collected in the piloting of the PSRK.‡ Specifically, we report on findings concerning micro-level support from men in the pregnant women's lives. In the quantitative component, we collected data on emotional, physical, material and informational support. After a careful reading of the literature, we defined emotional support as verbal or non-verbal interactions that show a person they are cared for; physical support as tangible assistance, such as helping with chores; material support as providing the pregnant woman with money or possessions; and informational support as providing guidance, advice or useful information about pregnancy. The qualitative component used narrative interviewing. In narrative interviews, the researcher poses open-ended questions that encourage the participant to engage in storytelling about aspects of their lives.^38^ In this case, the participants were asked to narrate aspects of their lives that they felt made their pregnancy journey more difficult or easier.

### Ethics

Ethics clearance was obtained from Rhodes University Human Research Ethics Committee [Ref no. 2020-2692-469] on 13 November 2020. The Eastern Cape Department of Health Research Committee granted permission to conduct the research [Ref no. EC 202109 019] on 24 February 2021. We aligned our approach to minors with the Department of Health’s (2015) recommendations (see section 3.2.2.4.): parental/guardian consent was sought if the pregnant minor indicated that their parent/guardian knew about the pregnancy; if the parent/guardian did not know and the potential participant did not wish to inform them about the pregnancy, the participant was asked to speak to a trusted adult about their participation in the study. This aligns with South African abortion law, which allows minors to consent to a termination of pregnancy without parental/guardian consent.

### Sampling

We administered the PSRK in two public health districts in the Eastern Cape – Amathole and Buffalo City. South Africa currently has a bifurcated healthcare system, with most of the population accessing care from the generally under-resourced and over-subscribed public sector.^39^ Antenatal care is free in this sector. Private healthcare, which is better resourced, is generally accessed by those on medical aid (health insurance) or those who can afford the out-of-pocket expenses (including antenatal care).

The two (out of seven) health districts were selected based on differing demographic composition and current maternal mortality to create a diverse sample. Buffalo City is largely urban, while Amathole is largely rural, with a few towns. The Eastern Cape has the highest maternal mortality in-facility rate in South Africa at 124.3 maternal deaths per 100,000 live births in the financial year 2022/2023. Buffalo City, the more urban district, has a maternal mortality in-facility rate of 164.6 maternal deaths per 100,000 live births, and Amathole, the more rural district, has 44.2 deaths per 100,000 live births (as recorded in the financial year 2022/2023).^40^

With the support of the two districts’ health managers, six antenatal clinics were selected in each district based on their volume of pregnant health users. Consecutive sampling was used to sample women in the clinics for both the quantitative and qualitative components. We trained antenatal healthcare workers in the ethical process of recruiting participants. The health workers informed all their pregnant health users about the study and asked whether they would like to participate. They handed out a leaflet in which the following was explained: the aim of the research, the researchers involved, the possible benefits and risks of participating, steps to ensure anonymity, ethics approval, and that (non)participation would not affect the services they receive. If participants agreed, the health workers took down their phone numbers and sent them to the research team.

The G*Power software (Version 3.1.9.7)^41^ was used to estimate an appropriate sample size for the quantitative component of this convergent mixed methods design. Chi-square test of independence was specified as the statistical model, with parameters set at *α* = 0.05, power (1-*β*) = 0.80, and a medium effect size (*w* = 0.20). This calculation suggested that a minimum sample size of 191 would ensure sufficient statistical power to address the research objectives effectively and to account for potential data loss (e.g. incomplete or missing responses). The objectives for this component were to ascertain the extent of perceived support reported across emotional, physical, material and informational domains (none, somewhat, very) reported by pregnant women in relation to partners and other significant men. Although the sample size calculation was based on a Chi-squared test of independence for categorical ratings of perceived support, the subsequent analyses were conducted with Generalised Estimating Equations models and McNemar’s test, both of which also address categorical outcomes.

The participants of the qualitative component were sampled from the list of willing participants sent by the health workers to the research team, with an even distribution across the two districts (20 in Amathole and 20 in Buffalo City). As no other demographic information was available at the time, the selection for the qualitative component within each district was random (i.e. randomly selected from the full list of potential participants sent by the health workers for the quantitative and qualitative components).

### Data collection

Data were collected via a telephonic survey for the quantitative component. Ten trained Black§ isiXhosa-speaking women fieldworkers telephoned the potential participants to obtain full verbal consent and administer the questionnaire. One fieldworker was present on each phone call, and the second author supervised the fieldwork . Participants received a token of appreciation for R50 airtime sent directly to their phones. Of those approached, 558 participants agreed to participate, 192.15% higher than the sample size recommended by the power analysis for the convergent mixed methods design. Of the 558 administered questionnaires, 106 were not used (deemed not applicable due to ineligible or incomplete responses), resulting in a data loss of 19.0% (106/558). Responses were recorded electronically, with 10% of interviews audited to ensure data quality.

Interviews for the qualitative component, generally one hour long, were conducted in the participant's language of preference (isiXhosa or English). A Black, isiXhosa-speaking woman conducted the interviews telephonically. The interviews were scheduled according to the participants’ convenience and ability to maintain privacy during the call. Narrative interviewing was used with the following as an initial prompt:

“*Please tell me about your pregnancy journey, from when you became pregnant until now. You can start anywhere you like. Please tell me about the things that made your pregnancy easy and those that made it difficult. I just want to hear your story, so I will listen. You can start when you are ready.*”

After listening to the initial narrative, the interviewer posed questions relating to the supportability framework elements, if they were not covered in the initial narrative (e.g. “Tell me about the support you received from men in your household”).

### Data analysis

Descriptive statistics were computed to summarise the distribution of support types (emotional, physical, material, and informational) received from partners. These statistics were stratified by gender (men and women) and household composition (same household or outside household) to provide a comprehensive overview of support patterns across demographic groups. To adjust for demographic imbalances between the two health districts from which participants were recruited, region-based weights were calculated to align the sample distribution with the estimated population distribution of pregnant women across the two regions. These weights were incorporated into all analyses conducted, ensuring that both percentages and model estimates more accurately reflect the underlying population structure.

To evaluate group differences in perceived support provision, we employed generalised estimating equations (GEE) models with a binomial distribution and logit link. GEE was selected because each participant reported on multiple types of support from different individuals in their social networks, creating correlated responses within participants. This approach appropriately accounts for such repeated-measures data using a working correlation structure. Odds ratios with 95% confidence intervals were estimated to facilitate the interpretation of differences across support types and relationship status. For comparisons of support provision from male versus female non-partners within the same relational category, McNemar’s Chi-squared tests with continuity correction were conducted. This test was chosen because the data were paired, with each participant providing responses about both male and female non-partners in the same category, making McNemar’s test the appropriate method to assess within-participant differences in proportions. All analyses were conducted using SPSS version 29.0.

The qualitative interviews were transcribed and translated by two different bilingual researchers, and the quality was checked by a third. The data were analysed using template analysis. Template analysis is a form of analysis in which researchers begin with *a priori* codes from either a subset of data or an existing theory.^42^ The *a priori* codes used came from the PSRK, and centred various forms of support (as noted above) provided by the various groups of people identified. Within the *a priori* codes, additional themes (coherent sets of meanings) are developed inductively. This analysis was conducted by the second author, with the team checking interpretations. Real names were replaced with pseudonyms, and all data were anonymised.

### Researcher positionalities

Data were collected in both components (qualitative and quantitative) by Black isiXhosa-speaking women to create a level of comfort for the participants. All three authors are senior South African academics at Rhodes University. The first and third authors are psychologists, and the second is a political scientist. The first two authors are White women, and the third author is a Black man. The second author oversaw the process of data collection, supporting and debriefing the fieldworkers as necessary. The three authors conducted the analysis with input from our research unit colleagues, consisting of a mix of Black and White, but mostly women, researchers, during work-in-progress colloquia, thereby contributing to trustworthiness. We were sensitive to the complexities of gendered and racialised positionalities in relation to the interpretation and discussion of findings. The in-depth discussions engaged in during work-in-progress sessions ensured that our interpretations were grounded in the social realities of the particular context.

## Findings

Of the 558 questionnaires that were answered, 241 were from the Amathole district, and 317 were from Buffalo City. After data cleaning, 192 responses from the Amathole district and 260 from Buffalo City were retained for analysis.

Table 2 outlines pertinent demographic information of these participants. Of note is that only 16.7% of our sample were married at the time of data collection. This is lower than the national statistics. Census 2022 data estimates that 37% of the population over 15 years old are legally married.^43^ The low marriage rate in the country is partially linked to men struggling to pay customary bridewealth, known as *lobola*, in contexts of high unemployment.^44^ Fewer participants were in a marital relationship than those with no partner at all.

**Table 2.** Pertinent participant demographic information for the quantitative component

Age	Pregnancy trimester	Education	Employment status	Relationship status
Range: 14–46	1st: 11.8%	Primary	Unemployed: 73.7%	Non-cohabiting partner: 44.8%
Mean: 27.8	2nd: 35.3%	Secondary: 82%	Employed: 26.3%	Co-habiting partner: 20.7%
	3rd: 52.8%	Higher		No partner: 7.8%
				Co-habiting married: 14.9%
				Non-cohabiting married: 1.8%

The interviewees in the qualitative component ranged in age from 18 to 38, with a mean age of 26.5 years. Five participants were in the first trimester of their pregnancy, 18 in the second, and 17 in the third. No other demographic data were collected for these participants.

We present findings on partner support and non-partner support below. In each section, we outline the results from the quantitative portion of the study first and follow this with insights from the qualitative portion.

### Partner support

In the quantitative component, 18.7% of those with partners indicated that their male partners provided some support, while 69.2% indicated that they received a lot of support. This means that just over one in ten participants (12.1%) felt they received no support *at all* from their partners during their pregnancy. Below, we outline results from the quantitative component and findings from the qualitative component in relation to “no support”, “some support”, and “a lot of support” responses.

#### No partner support: quantitative results

As outlined in Table 3, a few participants in the quantitative component reported no emotional or material support from their partners (4.2% and 7.1% respectively). A higher percentage indicated that their partners did not provide physical support (13.1%), while one out of four (24.1%) indicated that their partner provided them with no informational support. This illustrates the gendered assumption that men do not know about pregnancy.

**Table 3.** No partner support

No support	(%)
No emotional support	4.2
No physical support	13.1
No material support	7.1
No informational support	24.1

#### No partner support qualitative: abandonment, paternity denial and abuse

In the qualitative data, lack of partner support seemed to be linked to the partner not accepting the pregnancy.

Extract 1

*“You know how men are. When you are unmarried, a person can deny the pregnancy even though he knows you are in a relationship with him only … So, it was my fear at the beginning that what if he would just say ‘No’ after so many years together, you see?”* (Liyabona, 30, 5th month**)

Extract 2

*“He was a good person before I got pregnant. He just changed when I fell pregnant. … He does not support me at all. I am alone. I feel like I created this child alone because he does not even ask how me and the baby are. He is just quiet.”* (Sesethu, 27, 5th month)

Extract 3

*“We had so much planned, that we'd settle down together, and if I got a proper job we'd move together, that type of thing. But after I found out I was pregnant, people were saying that he was rejecting me. And I also realised that. But prior to this, we were always on the same page. So, all the things people were saying made some sense to me – that this person's actions were that of someone who was rejecting me, the physical abuse, acting funny towards me, calling me names.”* (Liyema, 33, 9th month)

Liyabona refers to a documented phenomenon in South Africa, where the intimate partners of young and unmarried women may deny paternity.^45,46^ Although she posits that paternity denial is well-known – “You know how men are” – she also has personal experience where the father of her first child denied paternity and abandoned her. Sesethu does not refer to paternity denial, *per se*, but rather abandonment because of the pregnancy. Like Sesethu, Liyema refers to a change in their intimate partner's behaviour after the pregnancy. Instead of the abandonment experienced by Sesethu, though, Liyema speaks of abuse. Each suggests that their intimate partner did not accept or support the pregnancy.

The lack of partner support seemed to be sufficiently normalised among unmarried pregnant couples that preparing for the possibility of being abandoned was considered shrewd.

Extract 4

*“And also, like next year, if I'm going to get a job, you know, because I don't want to be dependent on someone else because you know how men are. Sorry, I don't mean to speak ill of men, but I feel like men are just, sometimes you can be very happy together to be like, ‘Oh, I'm going to support you’, and then when the baby is here it's something different, you know. It's not what you expected, so you'd rather be ready on your side so that you don't have to ask or beg anyone for anything, you know.”* (Nontle, 30, 4th month)

Extract 5

*“I am giving him the benefit of the doubt [but I] leave room for disappointment. If maybe things don't happen like that [him providing support as promised], I'd also try. Like I am also saving up, so I don't get disappointed.”* (Sindiswa, 19, 8th month)

Extract 6

*“I often think, ‘What would I do if this boyfriend abandoned me during pregnancy?’ It's things like that that bother me. However, I always tell myself that even if he were to abandon me, I'd be able to hustle for myself and take care of my child. I'd be able to work and be an independent woman.”* (Lindiwe, 28, 3^rd^ month)

Like Liyabona above, Nontle invokes “common knowledge” about “how men are”. Despite her caveat (not speaking ill of men), she portrays men in general as unreliable. With this knowledge, then, it is prudent to be “ready on your side”. Other than having what is needed, such readiness, according to Nontle, enhances her dignity – not having to “beg” for things. Sindiswa and Lindiwe speak to possible disappointment or abandonment in personal terms. In each case, they invoke self-sufficiency as a solution: saving money, hustling, working, and being an “independent woman”.

#### Partners somewhat supportive: quantitative results

Table 4 outlines responses in the quantitative component where participants indicated that their partners were “somewhat supportive”. The percentage of women indicating that their intimate partners were somewhat supportive during pregnancy is higher than those indicating no support in every category other than information.

**Table 4.** Partners somewhat supportive

Somewhat supportive	(%)
Emotionally	15.5
Physically	23.1
Materially	18.8
Informationally	17.2

#### Somewhat supportive qualitative: half-heartedness, inconsistency, variability and reluctance

The qualitative data revealed dynamics where support was variable, precarious and non-continuous. Partner presence and acceptance of the pregnancy did not necessarily mean ongoing or consistent support. Some participants described how, despite their partner's offers to be helpful, these were half-hearted or inconsistent.

Extract 7

*“He must be in the mood for him to do something. If, for instance, I went to him for a weekend, he'd offer to cook but you'll hear him again asking to be helped or ask that I finish the cooking for him. Or sometimes, he offers to clean, but I've observed that he first has to be in the mood to do something.”* (Luthando, 24, 7th month)

Extract 8

*“He is a typical Xhosa traditional man. When I ask him to bring me water, he will ask, ‘Can't you do that for yourself?’ His support comes in the form of reminding me what they said I must do in hospital. He will remind me to exercise, eat right and he will call me out when I'm doing something that I'm not supposed to do. That's how he shows his support to me.”* (Hlengiwe, 27, 9th month)

Extract 9

*“He only speaks when he feels like it, and I don't care. I don't take it to heart anymore. I have already told myself that the baby is mine. Kids are a woman's responsibility because there is nothing we can do about men. I don't even tell him about my visits to the clinic anymore.”* (Andisiwe, 25, 7th month)

Luthando uses two examples of domestic labour that are generally feminised (cleaning and cooking). She suggests that her partner's offer of help is superficial (he expects her to complete the task) and is only offered if he feels like it – rather than based on her need. Hlengiwe similarly indicates that her partner resists taking on domestic chores like fetching water, citing his being a “typical Xhosa traditional man” (i.e. patriarchal) as the reason. She describes her partner's informational support as regulatory, using “what they said I must do” and “call me out” to control her behaviour. Andisiwe reports that her partner's mood determines his communication with her. She feels powerless in this – “nothing we can do about men”. “Ownership” of the baby grants her some power, however, and she, in turn, restricts what she tells her partner (not communicating about clinic visits).

Participants also spoke to variation in the type of support provided. Support in one area did not necessarily translate into support in another.

Extract 10

*“I told him that support is not only giving someone money. Support also comes in the form of emotional support because that is important also. I told him that I did not struggle a lot this month because of his support because he is calling me every night. Sometimes he sees me online at night and asks why I'm not sleeping. I would say that I'm struggling to sleep, and he would stay up and listen to all the rubbish that I will be saying.”* (Hlengiwe, 27, 9th month)

Extract 11

*“He gives me money. A lot of money … . I would have arguments and physical fights with [partner]. He'd hit and kick me on the side of my belly. And one day, I fell and slipped and fell on a rock.”* (Zola, 33, 8th month)

Hlengiwe, who was abandoned by her partner multiple times during her pregnancy, leaving her destitute, says he did so because of money. She talks about how she explained to him that support does not always mean “giving someone money”. She provides examples of his calling her, his concern that she is not sleeping, and his listening to “all [her] rubbish”, which she sees as supportive. Zola's intimate partner has a wife in his home village. He stays with Zola in her shack when he is in the city, usually late on Saturdays. He supports her financially. However, he also beats her. This worries Zola, telling the interviewer how her mother died in a domestic abuse dispute.

Support could also vary throughout the pregnancy.

Extract 12

*“He was happy for a couple of months, but things changed after two months. He started acting funny. He would deny that the child is his and stuff. I'd say, ‘I live with you. How can you say the child isn't yours?’ That's who he's become … Most of the time, I stay alone. I don't have anyone to talk to. I'm always crying because two months after finding out I was pregnant, he started drinking heavily.”* (Esethu, 28, 8th month)

Esethu poignantly speaks to a change in her partner's behaviour during the pregnancy. He started to drink heavily and to deny paternity. This results in her being alone and “crying”.

#### Partners very supportive: quantitative results

Table 5 outlines responses in the quantitative component where participants indicated that their partners were “very supportive”. Most participants in the quantitative component indicated that their partners were very supportive. Emotional support features as the most reported form of strong partner support, and informational support features as the least.

**Table 5.** Partners very supportive

Very supportive	%
Emotionally	80.3
Physically	63.9
Materially	74.1
Informationally	58.7

#### Partners very supportive qualitatively: extra effort and advocacy

Participants in the qualitative component spoke about how their partners put in additional effort and advocated for them.

Extract 13

*“Okay, in the first three months, I would not do laundry or even cook. I did not do anything. When a man returns from work, you, as a woman, are supposed to cook so he can come home and eat. He is tired. He would get home, and I would be resting in bed and had not made food. He wouldn't even change his clothes [hmm] and would just cook … it is said that a pregnant woman must not drink acidic beverages … He even stopped drinking acidic beverages. He also drinks juice. The one that I drink.”* Nobomi (25, 8th month)

Extract 14

*“My husband is really happy and excited about it [the pregnancy]. Like every appointment that we've had, he's been there. And we even at some point, we even changed our gynaecologist because he wouldn't allow my partner into the scanning room. So, my partner was like, ‘No, I'm not having it. We can always go to another one. I always want to see my baby every day’. So, yes*.” (Sivuyile, 28, 4th month)

Nobomi indicates how pregnancy changed her husband's behaviour. He would come home from work and cook for her. He also stopped drinking soft drinks along with her to help her quit the habit of imbibing sugary drinks. Of interest here is the assumption that, under usual circumstances, cooking is a woman's job. Thus, for men to be viewed as physically supportive means breaking the protocols of traditional gender roles during pregnancy. Sivuyile relates how invested her partner is in the pregnancy process, wanting to be completely part of the healthcare journey. His identification with “my baby” underpins, it appears, his involvement in and support of the pregnancy.

#### Relationship status and support

As indicated in the method section, only 16.7% of the sample was married. Table 6 outlines partner status in relation to support.

**Table 6.** Perceived no support provision by support type and relationship status

Predictor	Odds Ratio (95% CI)	*p*-value
**Support type**		
Physical vs Emotional	1.68 (1.38–2.04)	
Material vs Emotional	2.77 (2.28–3.37)	
Informational vs Emotional	2.51 (2.06–3.06)	
**Relationship status**		
Married, not living together vs Married, living together	1.28 (0.88–1.86)	.187
Unmarried, living together vs Married, living together	1.42 (0.99–2.03)	.052
Unmarried, not living together vs Married, living together	1.32 (0.89–1.96)	.161
**Interactions (support × relationship)**	All non-significant	>.24

Note: Emotional support and married living together are reference categories. Model estimated using GEE with binomial distribution and logit link.

Relationship status showed no clear association with perceived support, and interaction effects between relationship status and support type were minimal, indicating that the pattern of perceived no support was consistent across marital and cohabitation categories. However, perceptions of no support provision varied by support type, with women more likely to report no support in physical (OR = 1.68, 95% CI: 1.38–2.04), material (OR = 2.77, 95% CI: 2.28–3.37), and informational domains (OR = 2.51, 95% CI: 2.06–3.06) compared with emotional support. Model-predicted probabilities reinforced these findings, with approximately 11% of women reporting no emotional support, compared with 17% for physical support, 25% for material support, and 22% for informational support. These predicted values differ from the raw observed percentages in Table 2 because they are adjusted for repeated measures and covariates within the GEE framework. Taken together, these results suggest that practical forms of support, particularly material and informational, were more often perceived as lacking during pregnancy, while emotional support was least likely to be absent.

### Non-partner support

We asked participants in the quantitative component about the support they received from men in the same household, male family outside the home, and male friends. We compared this to reports of support from women in the same circumstances.

Of these participants, 60.2% lived with women in the same household, 41.7% lived with men in the same household, 86.5% had female family members living outside the household, and 68% had male family members living outside their household. Table 7 outlines “no support” responses for these categories of people. In this section, we concentrate on “no support” as an unambiguous variable.

**Table 7.** Analysis of no support reports during pregnancy by gender of non-partners (in the same household, family outside the home, friends)

Relational Category	*N* respondents	Men: No Support *n* (%)	Women: No Support *n* (%)	McNemar's *χ*^2^(1)	*p*
Same household	168	79 (47.0%)	27 (16.1%)	43.35	< .001
Family outside the home	339	236 (69.6%)	156 (46.0%)	61.19	< .001
Friends	181	92 (50.8%)	42 (23.2%)	40.02	< .001

Pregnant participants reported their perceptions of support provision from male and female non-partners across three relational categories. Gender differences were evident in all categories, with male non-partners more often perceived as providing no support than female non-partners. In the same household category (*n* = 168), 47.0% of women reported no support from at least one male non-partner compared with 16.1% from female non-partners (McNemar’s *χ*^2^ = 43.35, *p* < .001). Among family outside the home (*n* = 339), 69.6% reported no support from male non-partners versus 46.0% from female non-partners (*χ*^2^ = 61.19, *p* < .001). For friends (*n* = 181), 50.8% reported no support from male non-partners compared with 23.2% from female non-partners (*χ*^2^ = 40.02, *p* < .001). Taken together, these findings indicate a consistent pattern across relational categories, whereby male non-partners were more frequently perceived as failing to provide support during pregnancy than female non-partners, highlighting potential gender disparities in social support networks during this period.

In line with this, participants in the qualitative component spoke at length about the support their mothers provide. Where pregnant women live with their parents, their mothers would reportedly take over their chores, talk with them about their feelings, give advice about the pregnancy, and provide them with money, food and other pregnancy-related needs within their means. For example, Andisiwe (25, 7th month), whose partner abandoned her, said, “*At least I have my mother*”, whom she described as her “*pillar of strength”.* Similarly, Thandiwe (19, 6) said of her mother, “*She's the most supportive person here*”. Funeka (19, 6) indicated that her father provided financial support, but she said, “*I can only talk to my mother and sister”*.

Some participants related their fathers’ lack of perceived support to their following rigid sociocultural norms.

Extract 16

*“[My dad] does not do it the modern way. He does it the old people way. A typical example was when, after he found out, he was like, ‘Oh, I must traditionally strengthen the family so that the baby can enter the right space’ because* ukuqinisa umuzi [strengthen the family]. * … it's a traditional thing. I don't know how it's done exactly, but he said we must strengthen the family to get rid of dark spirits and all that. So, he does it his own way. And then my sister is like a 2000, not a 2000, but she's young. She's very google.”* (Nontle, 30, 4th month)

Extract 17

*“My father was angry. Even now, he is still not okay. He said he wanted cows before [marriage]. Old-fashioned people believe that you should get married first before having a child. The person who is supportive is my mother. And my grandmother.”* (Sesethu, 27, 5th month)

Nontle and Sesethu describe their fathers as “traditional” and “old-fashioned”; these men insist that particular rituals must be followed. Nontle, whose mother passed away, speaks of how much support she receives from her sister, who, she indicates, is modern. Like Nontle, Sesethu contrasts the lack of support from her traditional father with support provided by female family members, in this case, her mother and grandmother.

The expectation of motherly support was held up, even in the absence of a mother.

Extract 18

*“I was very worried, thinking that I might miscarry again. I was getting support, but I felt like I would have gotten better support from my mother. Maybe she would have given me good advice. … I always think that if my mother was still around, she would be giving me advice, and she would be helping me out in the house because my partner works far from home. He only comes back for four days.”* (Bathandwa, 30, 5th month)

Bathandwa talks about how the absence of her mother, who had passed away, affects her pregnancy. The suggestion is that she would worry less if her mother were around to give advice and help in the house.

In contrast, support expectations from male family members were low. Sisipho (23, 5th month) described how her grandmother brought her up and that she was not close to her father. She does not intend even to tell her father that she is pregnant since “*there is no need for him to know*” and that “*he'll just see the day he sees me*”. At times, simply not being critical or judgmental is considered to be support. For instance, Nobomi (25, 8th month) lives in a small town with her husband. When asked whether her father, who lives in Cape Town, is supportive, she responded, “*Yes, because he never shouted or did anything*”. When the interviewer asked why he would be angry at her, especially since she is married, she said, “*You know how men are. They like being feared*”.

In talk about friend support, female friends dominated. Support from male friends was often described as physical, such as pulling out chairs and helping with carrying things.

Extract 19

*“My friends, they are supportive in a way that they look out for me, you know? Sometimes, you know, when we feel like going out and everything, especially the guys, you know, the guys, they'd be quick to pull out chairs, you know, they’re quick to grab whatever you carry. They don't want me carrying stuff. And yeah, they try, they try, and they won't even say anything. They'll just take it [parcels] and leave, you know? [laugh] Or they'll just put a chair next to me and just look at me, you know? So, I know I'm expected to just sit down.”* (Sivuyile, 28, 4th month)

Extract 20

*“Yeah, they help me out a lot, you know. Especially those who have had a baby before. So, they have experienced everything that I'm experiencing, and they'll be like ‘No friend it's fine’. Like the other day, I was telling them like ‘Guys, it's weird and it's stressing me out that I really do not have an emotional attachment towards my growing baby. What is going on?’ They were like, ‘No, don't worry about it. It'll come around … just relax it will happen organically. It doesn't happen at the same time for everyone, but it will happen’.”* (Sivuyile, 28, 4th month)

In Extracts 19 and 20, Sivuyile contrasts support from male and female friends. The type of chivalrous behaviour she attributes to her male friends is consistent with masculine gender norms. While appreciating this kind of support, she also suggests that these male friends did not bother to ascertain her need or desire for such support. Instead, they “just take” parcels and “expect” her to sit down when they proffer a chair. In contrast, she describes female friends with children as emotionally and informationally supportive. She talks about being able to express her concerns, to which her female friends respond with reassurance.

Drawing these data together provides a clear picture of the gendered nature of the perceived support provided to pregnant women in our dataset. Table 8 summarises the percentages of participants who indicated that the relevant people provided no support at all (of any nature) during their pregnancy.

**Table 8.** Gendered picture of lack of support

No support provided (average)	%		%
Men		Women	
Partners	12.1		
Men same household	28.2	Women in same household	7.3
Men family outside household	56.7	Women family outside household	29.2
Men friends	34.3	Women friends	16.6

## Discussion

In this paper, we sought to understand the gendered dynamics appearing in women's reporting of, and stories concerning, support received during their pregnancy. Our concentration on gendered dynamics in women’s perceived support is in recognition of the fact that while male involvement in pregnancy can bolster women’s rights to reproductive health and bodily autonomy, it can also undermine these rights through their dominating decisions about the pregnancy or through their involvement not being welcomed or seen as supportive by women. Our inclusion of different types of support (emotional, physical, material, informational) moves the account beyond instrumental, clinic-based behaviours, which McLean^30^ argues is a “narrow” and “largely Western” definition of male involvement.

Our concentration on pregnancy support from a range of people in the pregnant person’s life extends existing research that tends to focus on, first, partners only, and second, partners’ experiences of, or involvement in, pregnancies. Given the fact that just under one-fifth of our sample indicated that they did not have a partner at the time of data collection, understanding the gendered dynamics of support beyond the intimate partner is important. Our research thus extends existing understandings of the gendered nature of pregnancy support to include men who are not pregnant women’s partners.

Although a majority of participants in the quantitative component indicated that they received a lot of support from their partners, the fact that three out of ten women indicated that they received no or only some support should be of concern. Our findings point to a number of gendered dynamics in partner support:

- Lack of partner support is underpinned by abandonment, paternity denial and abuse – to which women are particularly vulnerable if they are unmarried, young or have not formalised the relationship. Lack of partner support puts pressure on pregnant women to be self-sufficient, especially “hustling”, within an economically precarious environment.
- Where some support is provided, there are clear gendered dynamics, with men taking on domestic chores inconsistently or when in the “mood”.
- Informational support was rated very low in the quantitative component, and where such support was spoken about in the qualitative component, it was associated with warnings and judgement about the woman's behaviour and diet.

The quantitative component revealed that marriage and cohabitation had no effect on perceived support, except that unmarried partners not living together reported substantially higher levels of material support than their counterparts. Additionally, pregnant women may experience partner support in some areas and not others, and such support may shift during the pregnancy.

On the positive side, good partner support was associated with the partner identifying with and advocating for the pregnancy, changing habits in solidarity with healthy pregnancy behaviours, and taking on domestic chores. Emotional support was the most consistent form of perceived partner support reported in the quantitative component.

These findings cohere with research conducted elsewhere. Bottoman et al.'s study^47^ in the same province found that partner absence during pregnancy was seen as justified in non-marital contexts or when pregnant women were “moody”. Cumber et al.,^48^ in their review of partner involvement in pregnancies in Africa, suggest that traditional gender norms underpin variability of support, with men experiencing ridicule from their peers should they be seen to be supportive of their partners during pregnancy. Participants’ indication of men’s warnings and judgements aligns with Tokhi et al.’s^20^ observation that men’s involvement can result in their dominating decisions.

Beyond intimate partners, a distinct gendered pattern emerged, with men being seen as substantially less supportive than women. Male family members outside the household are reported as the least supportive during pregnancy, and women within the same household as the most. Across all categories, men are far more likely (at a statistically significant level) to be perceived as providing no support than their female counterparts. The burden of pregnancy support beyond the partner, therefore, falls on women. Mothers, in particular, were singled out as supportive, whereas fathers were reported as being “traditional”, holding up gendered norms. This gendered pattern is confirmed in Al-Mutawtah et al.'s review,^49^ in which female network connections were singled out as important in providing support during pregnancy. The support that men's network connections were said to provide – carrying parcels, pulling out chairs – while positive, indicates a comfortableness amongst men in performing traditional gender tasks (physical protection of women).

The repeated use of the phrase “you know how men are” in the qualitative dataset (featured three times in the short extracts presented in this paper) points to an assumption of shared understandings about gendered dynamics. The participants, speaking to a Black woman, invoke shared knowledge of the entrenchment or normalisation of gendered patterns of behaviour, suggesting that women have no choice but to navigate their way around “how men are”.

The limitation of this study is that we recruited women who access antenatal care in one province of South Africa. The gendered dynamics underpinning male support during pregnancy may differ in other parts of South Africa and other countries. South African women accessing care through the private healthcare sector may experience male support during pregnancy differently from those in our study. Men might view their behaviours as more supportive than is portrayed by women. Further exploration around the cultural/traditional and organisational limitations to male support would be useful, including how institutional and organisational support can invite men into the space of pregnancy care.

The use of non-random sampling in the quantitative component of this convergent mixed methods study introduces some limitations. For instance, selection bias may reduce generalisability, as certain demographic groups (e.g. those more willing to participate) could be over-represented. Non-random sampling also limits the transferability of integrated results to other populations or contexts. To compensate for a non-probability sampling strategy, we oversampled to increase representation, and weighting techniques were applied in the analysis to adjust for demographic imbalances. Nonetheless, external validity remains constrained. These results should also be interpreted within the context of repeated measures, considering that participants provided information on multiple support sources and types, which may introduce dependencies in the data structure.

In conclusion, from a public health perspective, men's support during pregnancy should be encouraged for its clear benefits and for enhancing women’s rights to reproductive health and well-being. Such support is, however, deeply imbricated in gendered dynamics. Abuse, abandonment and paternity denial may result in women having to garner social support elsewhere, generally from other women, in particular mothers. Even where support is provided, it may reinforce gendered norms (e.g. the feminisation of domestic chores) or be used to dominate decisions or regulate women's behaviours (e.g. judgemental reminders of antenatal care instructions).

Our findings reinforce other scholars’ appeals for nuance in understanding masculinities in relation to pregnancy.^9^ A simple public health message that male partner involvement in pregnancy is important fails to locate such support within the gendered norms and structural constraints operating within the specific context. While such messaging may assist in cases where pregnant women experience no support and abandonment, our research shows that gendered dynamics may play themselves out within the categories of some and even a lot of support. These dynamics include reinforcing that domestic chores are women’s work (although they may be suspended temporarily during pregnancy), that childcare is primarily women’s responsibility, that men can control women’s behaviour (e.g. what they eat during pregnancy), and that men should act as protectors (even when not requested to do so). Attending to these dynamics speaks to the rights of pregnant women, not only to reproductive health and bodily autonomy, but also to gender equity, a key goal within the global Sustainable Development Goals.

A nuanced understanding of gendered dynamics in men’s support of women during pregnancy is needed in multiple spaces. The antenatal care interaction, where male partners may experience themselves uncomfortably as neither patient nor visitor,^9^ is one such space. Equally important, however, is the social communication space within which men operate. Edgley and Roberts^50^ note, for example, that the advice given in published pregnancy guides written by men for men buttresses hegemonic masculine tropes, particularly stoicism, protectionism and rationality.

This is why the work of organisations such as Sonke Gender Justice (in South Africa) and MenEngage (international), referenced in the introduction, is so important. These bodies aim to work with men with the aim of “ending patriarchal power and supporting women's rights.”^10^ The aim of ensuring that pregnant women enjoy the right to non-discrimination and equality in relation to pregnancy support can be promoted through these sorts of organisations. For example, the MenCare programme run by Sonke Gender Justice tends to focus on fathers as caregivers for children and men’s presence in antenatal and perinatal spaces. Extending the content and conversations promoted by the programme to include the kinds of gendered dynamics that surfaced in this paper in relation to pregnancy support would complete the picture to promote gender equity in relation to reproduction and childbearing.

## Supplementary Material

Supplementary material: Relevant elements of the interview schedule.

Supplementary material: Elements of the PSRK used in this research.

## Footnotes

* Our research concentrates on cisgender men and women in heterosexual relationships.

** The first number refers to the age of the participant, the second to the gestational month of the pregnancy.

† Cognitive interviewing is a qualitative survey development method used in questionnaire design. The aim is to explore respondents’ understanding of survey items and to adjust as necessary to aid in item clarity [31].

‡ The team is in the process of refining the PSRK based on the pilot study. None of the questions that generated the data for this paper have been altered.

§ We use the term Black in the Black consciousness sense, including all people designated outside the racial category of White during the apartheid era. Most of our participants were Black African.
