# Supplementary material for: “You know how men are”: the gendered nature of support during pregnancy in South Africa – an exploratory convergent mixed-method study
Source: Sex Reprod Health Matters. 2026 Jun 17;33(1):2689806. doi: 10.1080/26410397.2026.2689806 (PMC13366643; doi:10.1080/26410397.2026.2689806)
Supplement: Supplementary material: Relevant elements of the interview schedule. [file ZRHM_A_2689806_SM4661.docx]

## Appendix: Relevant elements of the interview schedule

**INTERVIEW SCHEDULE**

Participant Identifier: ____________________________________

Interviewer: ____________________________________

Please tell me about your pregnancy journey, from when you became pregnant until now. You can start anywhere you like. Could you please tell me about the things that made your pregnancy easier and those that made it more difficult? I just want to hear your story, so I will listen. You can start when you are ready.

Age: ____________________________________

Month of pregnancy: ____________________________________

**PROBING QUESTIONS**

**SUPPORT: Partner; biological father; non-biological father**

**Please tell me about…**

| financial support | emotional support | physical support | informational support |
| --- | --- | --- | --- |
|  | | | |
|  | | | |
|  | | | |
|  | | | |
|  | | | |
|  | | | |

**SUPPORT: People living in your household**

**Please tell me about…**

| financial support | emotional support | physical support | informational support |
| --- | --- | --- | --- |
| Care for children | Provide care work | Need care work |  |
|  | | | |
|  | | | |
|  | | | |
|  | | | |
|  | | | |
|  | | | |
|  | | | |

**SUPPORT: Parents and other family members**

**Please tell me about…**

| financial support | emotional support | physical support | informational support |
| --- | --- | --- | --- |
|  | | | |
|  | | | |
|  | | | |
|  | | | |
|  | | | |
|  | | | |
|  | | | |

**SUPPORT: Friends; female friends; male friends**

**Please tell me about…**

| financial support | emotional support | physical support | informational support |
| --- | --- | --- | --- |
|  | | | |
|  | | | |
|  | | | |
|  | | | |
|  | | | |
|  | | | |
|  | | | |
