# Supplementary material for: “You know how men are”: the gendered nature of support during pregnancy in South Africa – an exploratory convergent mixed-method study
Source: Sex Reprod Health Matters. 2026 Jun 17;33(1):2689806. doi: 10.1080/26410397.2026.2689806 (PMC13366643; doi:10.1080/26410397.2026.2689806)
Supplement: Supplementary material: Elements of the PSRK used in this research. [file ZRHM_A_2689806_SM4660.docx]

## **Appendix: Elements of the PSRK used in this research [the full PSRK is undergoing refinement and will be available at a later date]**

1. How old are you?
   - 12-15
   - 15-19
   - 20-24
   - 25-29
   - 30-34
   - 35-39
   - 40+
2. What is the highest level of education you completed? Mark only one oval.
   - No schooling / Andina sikolo
   - Primary school (Grade 1 - 7)
   - Secondary school (Grade 8 - 12)
   - Matric Grade 12 completed
3. In which month of your pregnancy are you?

**Micro-Level: Partner Interaction**

1. Do you currently have a partner with whom you are in an intimate relationship?
   - Yes
   - No (Skip to question 14)
2. What kind of relationship is it? Mark only one oval.
   - Married and living with them
   - Married, but not living with them
   - Unmarried and living with them
   - Unmarried partner and not living together
3. Is your current partner the biological father?
   - Yes
   - No
4. In terms of emotional support, how supportive have your current partner been during this pregnancy? (i.e., talking and listening, showing care and compassion) Mark only one oval.
   - They are very supportive
   - They are somewhat supportive
   - They provide no support
5. In terms of physical support, how supportive have they been during this pregnancy? (i.e., carry things, rub feet, do household chores) Mark only one oval.
   - They are very supportive
   - They are somewhat supportive
   - They provide no support
6. In terms of financial and material support, how supportive have they been during this pregnancy? (i.e., give money, buy or give necessities) Mark only one oval.
   - They are very supportive
   - They are somewhat supportive
   - They provide no support
7. In terms of informational support, how supportive have they been during this pregnancy? (Give advice and guidance) Mark only one oval.
   - They are very supportive
   - They are somewhat supportive
   - They provide no support
8. Has your partner ever denied paternity? Mark only one oval.
   - Yes
   - No
   - I don't want to say
9. Has your partner ever abandoned you during the pregnancy? Mark only one oval.
   - Yes
   - No
   - I don't want to say
10. Has your partner ever physically, emotionally or sexually abused you? Mark only one oval.
    - Yes
    - No
    - I don't want to say

**Micro Level: Household size**

1. How many people normally live in your household with you? Mark only one oval.
   - 0
   - 1
   - 2
   - 3 or more
2. How many of these people are children? Mark only one oval.
   - 0
   - 1
   - 2
   - 3 or more

**Micro Level: Women in same household as you**

1. Are there women living in the household with you? Mark only one oval.
   - Yes
   - No (Skip to question 21)
2. In terms of emotional support, how supportive have they been during this pregnancy? (i.e., talking and listening, showing care and compassion) Mark only one oval.
   - They are very supportive
   - They are somewhat supportive
   - They provide no support
3. In terms of physical support, how supportive have they been during this pregnancy? (i.e., carry things, rub feet, do household chores) Mark only one oval.
   - They are very supportive
   - They are somewhat supportive
   - They provide no support
4. In terms of financial and material support, how supportive have they been during this pregnancy? (i.e., give money, buy or give necessities) Mark only one oval.
   - They are very supportive
   - They are somewhat supportive
   - They provide no support
5. In terms of informational support, how supportive have they been during this pregnancy? (Give advice and guidance) Mark only one oval.
   - They are very supportive
   - They are somewhat supportive
   - They provide no support

**Micro Level: Men in same household as you**

1. Are there men living in the same household as you? Mark only one oval.
   - Yes
   - No (Skip to question 26)
2. In terms of emotional support, how supportive have they been during this pregnancy? (i.e., talking and listening, showing care and compassion) Mark only one oval.
   - They are very supportive
   - They are somewhat supportive
   - They provide no support
3. In terms of physical support, how supportive have they been during this pregnancy? (i.e., carry things, rub feet, do household chores) Mark only one oval.
   - They are very supportive
   - They are somewhat supportive
   - They provide no support
4. In terms of financial and material support, how supportive have they been during this pregnancy? (i.e., give money, buy or give necessities) Mark only one oval.
   - They are very supportive
   - They are somewhat supportive
   - They provide no support
5. In terms of informational support, how supportive have they been during this pregnancy? (Give advice and guidance) Mark only one oval.
   - They are very supportive
   - They are somewhat supportive
   - They provide no support

**Micro Level: Women family members outside the same household as you**

1. Do you have women family members who live outside of the same household as you? Mark only one oval.
   - Yes
   - No (Skip to question 31)
2. In terms of emotional support, how supportive have they been during this pregnancy? (i.e., talking and listening, showing care and compassion) Mark only one oval.
   - They are very supportive
   - They are somewhat supportive
   - They provide no support
3. In terms of physical support, how supportive have they been during this pregnancy? (i.e., carry things, rub feet, do household chores) Mark only one oval.
   - They are very supportive
   - They are somewhat supportive
   - They provide no support
4. In terms of financial and material support, how supportive have they been during this pregnancy? (i.e., give money, buy or give necessities) Mark only one oval.
   - They are very supportive
   - They are somewhat supportive
   - They provide no support
5. In terms of informational support, how supportive have they been during this pregnancy? (Give advice and guidance) Mark only one oval.
   - They are very supportive
   - They are somewhat supportive
   - They provide no support

**Micro Level: Men family members outside same household as you**

1. Do you have men family members outside same household as you? Mark only one oval.
   - Yes
   - No (Skip to question 36)
2. In terms of emotional support, how supportive have they been during this pregnancy? (i.e., talking and listening, showing care and compassion) Mark only one oval.
   - They are very supportive
   - They are somewhat supportive
   - They provide no support
3. In terms of physical support, how supportive have they been during this pregnancy? (i.e., carry things, rub feet, do household chores) Mark only one oval.
   - They are very supportive
   - They are somewhat supportive
   - They provide no support
4. In terms of financial and material support, how supportive have they been during this pregnancy? (i.e., give money, buy or give necessities) Mark only one oval.
   - They are very supportive
   - They are somewhat supportive
   - They provide no support
5. In terms of informational support, how supportive have they been during this pregnancy? (Give advice and guidance) Mark only one oval.
   - They are very supportive
   - They are somewhat supportive
   - They provide no support

**Micro Level: Female friends**

1. Do you have female friends? Mark only one oval.
   - Yes
   - No (Skip to question 83)
2. In terms of emotional support, how supportive have they been during this pregnancy? (i.e., talking and listening, showing care and compassion) Mark only one oval.
   - They are very supportive
   - They are somewhat supportive
   - They provide no support
3. In terms of physical support, how supportive have they been during this pregnancy? (i.e., carry things, rub feet, do household chores) Mark only one oval.
   - They are very supportive
   - They are somewhat supportive
   - They provide no support
4. In terms of financial and material support, how supportive have they been during this pregnancy? (i.e., give money, buy or give necessities) Mark only one oval.
   - They are very supportive
   - They are somewhat supportive
   - They provide no support
5. In terms of informational support, how supportive have they been during this pregnancy? (Give advice and guidance) Mark only one oval.
   - They are very supportive
   - They are somewhat supportive
   - They provide no support

**Micro Level: Male friends**

1. Do you have male friends? Mark only one oval.
   - Yes
   - No (Skip to question 88)
2. In terms of emotional support, how supportive have they been during this pregnancy? (i.e., talking and listening, showing care and compassion) Mark only one oval.
   - They are very supportive
   - They are somewhat supportive
   - They provide no support
3. In terms of physical support, how supportive have they been during this pregnancy? (i.e., carry things, rub feet, do household chores) Mark only one oval.
   - They are very supportive
   - They are somewhat supportive
   - They provide no support
4. In terms of financial and material support, how supportive have they been during this pregnancy? (i.e., give money, buy or give necessities) Mark only one oval.
   - They are very supportive
   - They are somewhat supportive
   - They provide no support
5. In terms of informational support, how supportive have they been during this pregnancy? (Give advice and guidance) Mark only one oval.
   - They are very supportive
   - They are somewhat supportive
   - They provide no support

**Micro Level: Employment and work support**

1. Please tick the box most relevant to your employment situation. Mark only one oval.
   - I am employed
   - I am not employed
